# Safety Events Associated with Tramadol Use Among Older Adults with Osteoarthritis

**DOI:** 10.1089/pop.2019.0220

**Published:** 2021-02-02

**Authors:** Shirley Musich, Shaohung S. Wang, James A. Schaeffer, Luke Slindee, Sandra Kraemer, Charlotte S. Yeh

**Affiliations:** ^1^ Research for Aging Populations, Optum, Ann Arbor, Michigan, USA.; ^2^ Optum Enterprise Analytics, Eden Prairie, Minnesota, USA.; ^3^ UnitedHealthcare Medicare & Retirement, Minneapolis, Minnesota, USA.; ^4^ AARP Services, Inc., Washington, District of Columbia, USA.

**Keywords:** tramadol, safety events, falls, hospitalizations, mortality

## Abstract

Tramadol is a low-level opioid increasingly recommended to treat moderate-to-severe acute and chronic pain. Although characterized as having fewer opioid-related adverse events, the longer term safety of tramadol use among older adults has not been thoroughly documented. Thus, the primary objective was to examine the risk of safety events associated with chronic tramadol use compared to other chronic opioid use or no opioids among older adults with osteoarthritis. Safety events considered included: ≥3 emergency room (ER) visits, falls/hip fractures, cardiovascular (CVD) hospitalization, composite safety event hospitalization, and all-cause mortality. The study population included older adults ages ≥65 years diagnosed with osteoarthritis and classified into new or continuing tramadol use, new or continuing other opioid use, or nonuse. Inclusion criteria included: 6-month pre period and up to 33 months post period. Tramadol, other opioid, and no opioid users were 1:1 propensity-matched providing study populations of 25,899 within each category; 72% were new chronic opioid users. Multiple logistic regression or Cox proportional hazard ratios were used to document risk. Generally, tramadol users had fewer adverse event risks compared to other opioid users but higher risks than nonusers. New users of tramadol or other opioids had higher risks than continuing users. Tramadol use was associated with increased risk of multiple ER utilizations, falls/fractures, CVD hospitalizations, safety event hospitalizations, and mortality (new users only) compared to nonuse. Thus, although tramadol use may be appropriately recommended within a pain management strategy for older adults with osteoarthritis, careful monitoring for adverse safety events is warranted.

## Introduction

Tramadol is a low-level opioid increasingly recommended for the treatment of moderate-to-severe acute and chronic pain when acetaminophen and/or nonsteroidal anti-inflammatory drugs (NSAIDs) no longer provide adequate analgesic effectiveness.^1–5^ As of 2012, the American College of Rheumatology and the American Academy of Orthopedic Surgeons updated their guidelines to recommend tramadol as a first-line treatment option, along with NSAIDs, for osteoarthritic pain.^6^ Furthermore, compared to other opioids, the US Drug Enforcement Agency (DEA) has ranked tramadol as having the lowest risk of dependence or addiction among categories of opioids.^7^

Adding to tramadol recommendations, 10-day and 12-week clinical trials documented reduced levels of opioid-related adverse events, including constipation, respiratory distress, and somnolence.^3–5^ Conclusions from these trials indicated that, while the potential for selected adverse events was reduced, use of tramadol was not without risk for similar levels of dizziness, nausea, and headaches compared to other stronger opioids.

Contraindications associated with tramadol listed by the US Food and Drug Administration (FDA) include: prolonged elimination half-life for patients ages >75 years with recommended adjustment in daily dosages; a risk of serotonin syndrome and seizures, especially in combination with antidepressants; respiratory distress with high doses; and interactions with other central nervous system (CNS) depressants (eg, tranquilizers, sedative hypnotics).^8^ In addition, the FDA warns that tramadol may impair mental and/or physical abilities and cautions the performance of hazardous tasks such as driving.^8^ Despite these potential adverse effects, given the proven analgesic properties of tramadol,^3–5^ low risk of dependence,^7^ and gastrointestinal, renal, and cardiovascular warnings associated with chronic NSAID use,^1,2^ physicians have increasingly relied on tramadol as a pain management solution, especially for chronic osteoarthritic or musculoskeletal pain.^9–12^

Although clinical trials have documented short-term adverse effects of tramadol use^3–5^ and contraindications listed by the FDA^8^ have highlighted risks associated with tramadol use, fewer published research studies have examined longer term adverse safety events associated with tramadol use. These longer term safety events have included emergency room (ER) utilization,^9^ drug-related hospitalizations,^10^ falls/fractures,^13,14^ and all-cause mortality.^11,15^ Most research studies have focused on risks associated with overall opioid use.^16–19^ Furthermore, despite indications of potential risks, many primary care providers have assumed this drug to be relatively safe compared to other pharmaceutical options for patients vulnerable to adverse events, especially older adults.^15^

Diagnostic documentation of some adverse events, such as seizures, respiratory distress, non-injurious falls, or other adverse drug-drug interactions, are difficult to track in administrative databases because of insufficient coding detail. However, ER utilization can serve as a surrogate measure for these lower-level events.

In one surveillance report, tramadol-related ER visits increased as tramadol use increased between 2005 and 2011.^9^ About 60% of visits were associated with tramadol only; 40% in combination with other pharmaceuticals including other opioid pain relievers, cardiovascular medications and antidepressants. Older adults (ages ≥65 years) were the largest age group (35%) associated with tramadol-related ER use. In another population-based study including those ages ≥18 years, tramadol was associated with a 52% increased risk of hospitalization for hypoglycemia with the highest risk evident at initiation.^10^ Other sources of safety event hospitalizations potentially associated with opioids/tramadol include respiratory distress,^8^ pneumonia,^20^ fractures,^14,16–19^ and seizures.^8,21–23^

The risk for injurious falls/fractures is consistently identified as an adverse event associated with overall opioid use, likely prompted by dizziness, fatigue, somnolence, and the other CNS effects of opioids.^3–5^ The risk for falls/fractures is higher with drug initiation and tends to dissipate over time to lower levels of risk.^13,14^ Fewer studies focus exclusively on the association of tramadol with injurious falls/fractures but, generally, the level of risk associated with tramadol (eg, odds ratio [OR] 1.54)^14^ compared to other opioids (eg, ORs 1.36–1.59)^14^ was similar.^13,17–19^

The association between opioids and cardiovascular (CVD) outcomes has not been widely reported and results have been mixed.^2,16,24,25^ In a recent study among those ages ≥45 years using subgroups of opioids, no significant association was evident between opioid medication use and coronary artery disease.^25^ A 2007 review summarizing the use of tramadol for management of chronic pain indicated that tramadol had no known associations with CVD adverse effects, gastrointestinal bleeding, or renal toxicity in contrast to contraindications associated with chronic NSAIDs.^2^ Nevertheless, a 77% increased risk of specific CVD events with opioid use was documented in a Medicare population of low income older adults ages ≥65 years.^16^ Although tramadol has not been specifically evaluated for CVD risks, the Solomon et al^16^ opioid evidence would indicate that this aspect of tramadol warrants additional attention.

As with other longer term outcomes, all-cause mortality has been associated primarily with more potent opioids and often focused on younger populations.^26^ However, in one study utilizing Medicare beneficiaries with osteoarthritis or rheumatoid arthritis, the risk for all-cause mortality was elevated by 87%.^16^ More recently, research studies have demonstrated a significant risk for all-cause mortality associated with tramadol use.^11,15^ Although the authors expressed caution interpreting the results related to the risk of residual confounding, the 2 studies reported similar levels of elevated risk—about 70% increased risk of all-cause mortality associated with tramadol.

Combining evidence from clinical trials, documentation from the DEA, and recommendations from provider organizations, common perceptions prevail that few or no adverse risks will be associated with chronic tramadol use.^15^ However, few tramadol research studies focused specifically on longer term safety event outcomes compared to other opioids or no opioid solutions among older adults.^11,15–17^ Consequently, it was of interest to systematically document selected longer term outcomes associated with chronic tramadol use in a population of older adults with osteoarthritis enrolled in a Medigap medical insurance plan.

In the United States, Medicare fee-for-service plans (about 70% of all Medicare plans) pay about 80% of medical expenditures for these individuals and offer minimal prescription drug benefits. Although most (about 90%) of those with original fee-for-service Medicare purchase additional insurance plans to cover the remaining 20% of medical expenses, about 28% (currently about 10.2 million adults) have purchased Medigap coverage.^27^ Because this population may differ from general older adult and/or overall Medicare populations, it was of interest to estimate the risk for longer-term safety events, such as ER visits, hospitalizations, falls/fractures, and/or mortality, associated with chronic tramadol use compared to other opioids or opioid nonuse. In addition, because most opioid studies focus on opioid initiation or new users,^10,11,13,16,17,19^ the present study included a comparison of new and continuing users to document the assumption that risks associated with tramadol or other opioid use would dissipate over time.^10,13,14,18^

The expectation was that tramadol would be associated with excess risk for safety events compared to no opioid use, and that the risks associated with tramadol would be less than those associated with other more potent opioids. New users were expected to have higher risks for adverse events compared to continuing users, and continuing users were expected to remain at higher levels of risk compared to non-opioid users.

Thus, the objective of this study was to estimate the risk of safety events associated with new or continuing chronic tramadol use compared to new or continuing other chronic opioid use or no opioid use among older adults with diagnosed osteoarthritis. Safety events included: multiple ER use (≥3), injurious falls/hip fractures, CVD hospitalizations, composite safety event (ie, CVD, pneumonia, respiratory distress, injurious falls/hip fractures and/or serotonin syndrome) hospitalizations, or all-cause mortality. This research was covered under the New England IRB #120160532.

## Methods

### Study population

In 2016, approximately 5 million Medicare insureds were covered by an AARP^®^ Medicare Supplement Insurance Plan insured by UnitedHealthcare Insurance Company (UnitedHealthcare Insurance Company of New York for New York certificate holders). These plans are offered in all 50 states, Washington, DC, and various US territories. AARP Medicare Supplement insureds with AARP MedicareRx plans from UnitedHealthcare (about 55% of insureds) had to have been at least 65 years of age with continuous medical and drug plan enrollment for a minimum of 6 months pre period (January-June 2016) and up to 33 months post period (June 2016-March 2019). In addition, insureds must have been newly diagnosed with degenerative joint disease (ie, osteoarthritis) prior to the opioid use. Those with cancer or sickle cell diagnoses were excluded.

### Defining new and continuing chronic tramadol users, other opioid users, and no opioid controls

Tramadol and other opioids were identified from National Drug Codes (NDCs) as recommended by the 2018 Healthcare Effectiveness Data and Information Set (HEDIS) quality measures associated with opioid use.^28^ New tramadol or other opioid users must have had a clean 6-month pre period with no tramadol or other opioid prescriptions, then initiated tramadol or other opioid use after their osteoarthritis diagnoses, and continued to use tramadol or other opioids for a minimum of 31 days (ie, chronic user). New tramadol users must have had a minimum of 75% tramadol use among their total opioid prescriptions.

Continuing tramadol or other opioid users had existing prescriptions for tramadol or other opioids during the 6-month pre period and continued using after their osteoarthritis diagnoses for a minimum of 31 days. Continuing tramadol users must have had at least 75% tramadol use among their total opioid prescriptions.

### Propensity score matching

Tramadol and other opioid users were more likely to be female, older, lower income, and to have had more chronic conditions than the non-opioid controls (data not shown). Consequently, 1:1 propensity matching was used to equalize the 6 cohorts: new tramadol, new other opioid, and no opioid control; and continuing tramadol, continuing other opioid, and no opioid control. Study populations were matched on age, sex, minority status, income, location, region, health status, and medical conditions (variables are listed in Table 1).^29,30^ The final study populations included 25,899 each for tramadol, other opioids, and no opioid control cohorts; overall, 72% were new users of either tramadol or other opioids and 28% were continuing users.

**Table 1.** Demographics Associated with Propensity Matched New and Continuing Tramadol, Other Opioid, and No Opioid Users: One-Year Follow-Up

Variables	New users				Continuing users			
	Tramadol % or mean	Other opioids % or mean	No opioid control % or mean	P	Tramadol % or mean	Other opioids % or mean	No opioid control % or mean	P
Number	18,824	18,661	18,824		7075	7238	7075	
Sex								
Male	29.7	30.1	28.5	0.003	28.0	27.5	27.1	0.48
Female	70.3	70.0	71.5		72.0	72.5	72.9	
Age (years)	77.0	76.8	77.1	0.0001	77.0	77.1	77.5	0.001
65–69	20.7	21.0	19.7	0.006	19.4	18.8	18.9	<0.0001
70–74	23.8	23.7	24.1		23.8	23.5	22.7	
75–79	20.3	21.3	20.6		22.8	21.9	21.3	
80–85	14.9	14.5	14.7		15.3	16.1	14.7	
≥85	20.3	19.6	20.9		18.6	19.7	22.4	
Minority								
Low	48.2	48.6	48.3	0.39	47.8	48.2	47.5	0.92
Medium	46.9	46.9	47.1		47.4	47.2	47.5	
High	3.3	3.3	3.1		3.4	3.3	3.5	
Income								
Low	16.6	16.2	16.2	0.29	17.0	17.9	16.7	0.41
Medium	36.9	36.9	36.4		37.4	37.8	38.3	
High	46.2	46.4	47.0		45.3	44.1	44.6	
Location								
Metro	83.9	84.4	84.9	0.02	84.4	82.9	84.3	0.02
Other	16.1	15.6	15.1		15.6	17.1	15.7	
Region								
Midwest	16.8	17.1	16.7	0.43	16.6	16.8	16.7	0.005
Northeast	19.8	19.8	19.5		18.5	19.2	17.2	
South	45.9	45.8	46.3		48.2	47.8	49.7	
West	17.1	16.9	17.0		16.5	16.0	15.8	
Access to health care								
PCPs per 100,000	130.2	130.3	129.7	0.19	129.4	129.5	128.0	0.02
Plan type								
High coverage	77.3	77.2	76.2	0.05	78.3	78.9	76.4	0.002
Medium coverage	2.7	2.7	3.0		2.4	2.7	2.5	
Other	20.0	20.1	20.8		19.4	18.4	21.0	
HCC score								
<0.50	20.5	21.9	20.0	0.0003	16.6	13.8	16.0	<0.0001
0.50 to <1.20	43.3	43.1	44.1		40.8	40.6	42.2	
1.20 to <2.80	29.6	28.7	29.5		34.5	36.3	33.8	
≥2.8	6.7	6.3	6.4		8.1	9.4	8.0	
EBM-C conditions								
Asthma	8.4	8.6	8.4	0.89	11.3	10.9	9.7	0.009
Atrial fibrillation	15.7	15.5	14.8	0.03	16.9	18.1	17.2	0.13
CAD	22.5	21.8	22.1	0.26	26.6	28.0	25.3	0.002
CHF	10.1	9.6	9.4	0.09	10.9	12.1	11.6	0.07
COPD	11.3	11.1	10.4	0.01	13.3	13.9	12.8	0.14
Depression	11.6	11.4	10.8	0.04	14.2	14.8	13.7	0.15
Diabetes	24.1	23.9	23.8	0.77	26.3	27.0	25.7	0.22
Hyperlipidemia	59.6	59.6	61.0	0.007	68.0	68.3	64.5	<0.0001
Hypertension	78.1	78.0	79.3	0.003	86.1	86.6	84.4	0.0005
Kidney disease	13.1	12.8	12.0	0.002	14.4	15.5	15.5	0.08
Obesity/overweight	20.5	20.8	20.5	0.79	24.8	24.3	24.3	0.73
Osteoporosis	10.9	10.9	11.1	0.69	13.9	13.4	12.2	0.01
Rheumatoid arthritis	4.4	4.5	4.4	0.83	6.7	6.2	6.2	0.39
Injury fall/hip fracture	13.0	14.7	6.3	<0.0001	11.4	12.9	6.9	<0.0001
Insomnia dx	9.8	11.4	5.6	<0.0001	10.0	12.7	5.7	<0.0001
Pneumonia dx	9.6	10.3	7.7	<0.0001	8.7	9.8	8.1	0.001
Serotonin syndrome dx	0.4	0.8	0.0	<0.0001	0.3	0.8	0.1	<0.0001
Respiratory distress dx	27.2	29.4	22.6	<0.0001	28.3	31.2	24.8	<0.0001
Other medication use								
Antidepressant Rx	40.0	43.5	28.1	<0.0001	43.4	49.2	29.6	<0.0001
Antipsychotics Rx	4.4	4.6	3.5	<0.0001	4.4	4.9	3.6	0.001
Benzodiazepine Rx	26.5	31.9	15.4	<0.0001	28.2	34.4	16.3	<0.0001
Gabapentinoids Rx	29.5	35.3	9.5	<0.0001	30.9	38.5	10.1	<0.0001
Muscle relaxant Rx	17.3	23.3	4.5	<0.0001	15.1	20.1	4.9	<0.0001
Non-benzo-hypnotics Rx	14.6	17.3	7.7	<0.0001	16.2	19.9	7.7	<0.0001
NSAID Rx	36.8	38.6	16.0	<0.0001	35.0	36.2	15.8	<0.0001
CNS Medications								
0	39.1	31.4	68.5	<0.0001	37.4	29.2	67.3	<0.0001
1	36.4	36.7	23.7		37.8	36.5	24.5	
2	18.4	22.0	6.4		18.6	23.7	6.7	
≥3	6.0	9.9	1.3		6.3	10.5	1.5	
Physical therapy	44.9	53.3	22.8	<0.0001	39.8	47.4	22.3	<0.0001
ER visits in 1 year								
0–2	87.1	84.1	93.5	<0.0001	88.6	84.5	93.2	<0.0001
≥3	12.9	15.9	6.5		11.4	15.5	6.8	
Months of follow-up	22.7	23.0	24.4	<0.0001	28.4	28.4	24.7	<0.0001

CNS medications include benzodiazepines, non-benzo hypnotics, muscle relaxants, antipsychotics and gabapentinoids. Missing were calculated separately but deleted for brevity.

CAD, cardiovascular disease; CHF, congestive heart failure; CNS, central nervous system; COPD, chronic obstructive pulmonary disease; dx, diagnoses codes; EBM-C, Evidence-Based Medicine-Connect software; ER, emergency room; HCC, Hierarchical Condition Category; NSAID, nonsteroidal anti-inflammatory drug; PCP, primary care physician; Rx, prescription.

### Covariates

Covariates were included to propensity match tramadol, other opioid, and no opioid controls, and to adjust for other factors. These covariates included measures of demographics, socioeconomic factors, health status, and other characteristics taken from health plan eligibility and administrative medical claims.

Demographic questions included age and sex. Age groups were defined as: 65–69; 70–74; 75–79; 80–84; and ≥85 years. Geographic regions (Northeast, South, Midwest, and West); location (metropolitan and other); low (<15% nonwhite), medium (15% to 59% nonwhite) and high (≥60% nonwhite) minority areas; and low (<$40,179), medium ($40,179 to <$ 57,199), and high (≥$57,199) median household income levels were geocoded from zip codes. AARP Medicare Supplement plan types were grouped by cost-sharing levels, including high-level coverage plans with minimal co-payments or deductibles, less comprehensive medium-level coverage, and all other plans. A health services access variable was calculated as primary care providers per 100,000 capita.

Level of medical services utilization from medical claims was calculated as the Hierarchical Condition Category (HCC) score.^31^ The HCC score is used by the Centers for Medicare & Medicaid Services to risk adjust medical payments across various medical plans according to the health status of the different insured populations. HCC subgroups were utilized to control for health status and defined as follows: HCC scores <0.5 (healthy and active); HCC scores 0.5 to <1.2 (above average); HCC scores 1.2 to <2.8 (at risk); and HCC scores ≥2.8 (very sick). Physical therapy sessions were identified from procedure codes not linked to any specific diagnosis codes.

### Common chronic conditions

Thirteen chronic conditions identified using Evidence-Based Medicine Connect software (Symmetry EBM Connect, version 9.2) were included in the propensity matching. This software was developed to measure quality of care from health care claims data, using a defined set of measures for evidence-based care associated with various medical conditions.

Other opioid-related medical adverse events were identified from diagnosis codes including: insomnia, respiratory distress, pneumonia, and serotonin syndrome.

### Other medication use

Other medications often used in combination with opioids to manage pain, insomnia, anxiety, or depression include: anti-inflammatory nonsteroidal drugs (NSAIDs), antidepressants, benzodiazepines, non-benzodiazepine hypnotics, muscle relaxants, antipsychotics, and gabapentinoids. These medications were identified using NDCs.

Combinations of the CNS-active medications were defined by counting selected CNS medications (benzodiazepines, non-benzodiazepine hypnotics, muscle relaxants, antipsychotics and gabapentinoids) and categorizing as: 0, 1, 2, and ≥3 of these medications. Antidepressants were assessed as a stand-alone category because of the documented serotonin syndrome adverse effect associated with the combination of tramadol and antidepressants.

### Injurious falls/hip fractures

Injurious falls requiring medical services or hip fractures, as a combined measure, were defined from suggested HEDIS diagnoses codes.^27^ Patients were followed until the injurious fall/hip fracture event or until the end of follow-up in March 2019.

### Multiple ER visits, CVD hospitalization, safety event hospitalization, and mortality

ER visits were identified from place of service codes along with procedure codes and revenue codes within medical claims. Based on the distribution, multiple ER visits were defined as ≥3 visits within the first year of follow-up.

CVD hospitalization patients were identified from *International Classification of Diseases* (ICD)-9 or ICD-10 diagnosis codes. Patients were followed until a CVD hospitalization or until the end of follow-up in March 2019.

Safety event hospitalizations was a combined measure including the following adverse events associated with a hospitalization: CVD hospitalization, injurious fall/hip fracture, pneumonia, respiratory distress, or serotonin syndrome seizure. Hospitalization events were identified from the ICD-9 or ICD-10 diagnosis codes documented in inpatient admission claims. Patients were followed until a hospitalization event or until the end of follow-up in March 2019.

All-cause mortality was determined from the AARP Medicare Supplement eligibility files maintained by UnitedHealthcare. Patients must have had a 6-month pre period and must have been alive with at least 12 months of plan eligibility; then were followed until death or until the end of follow-up in March 2019.

### Statistics

Demographic variables were statistically tested across the 3 new and 3 continuing tramadol, other opioid and no opioid categories using chi-square for categorical variables or *t* tests for continuous variables, considering *P* ≤ 0.05 as significant. All analyses were completed using SAS Enterprise Guide Version 7.1 (SAS Institute Inc., Cary, NC, USA). Missing demographic variables (income, minority, region, and location) were treated as separate categories, although because these variables were calculated from address zip codes, missing data were minimal (<1.5% on any one variable). Consequently no imputation of data was utilized.

Unadjusted multiple ER utilization, falls/hip fractures, CVD hospitalization, safety event hospitalization, and mortality rates by opioid use categories were determined. Subsequently, results were regression adjusted to control for any significant variable differences that may have remained after the propensity matching.

Logistic regression models were used to determine the risk of multiple ER utilization within 1 year of follow-up associated with tramadol or other opioids compared to no opioid controls, adjusted for variables listed in Table 2. Because ER visits are the summation of multiple ER events, the time frame utilized for these analyses was limited to 1 year. ER visits were used to capture drug-drug interaction events and other less serious adverse events that are not regularly coded within the medical claims.

**Table 2.** Regression Adjusted Odds Ratios for Risk of ≥3 Emergency Room Visits Associated with Tramadol and Other Opioids Versus No Opioids

Variable	New user		Continuing user	
	Odds ratios	P	Odds ratios	P
**Tramadol**	**1.8**	**<0.0001**	**1.5**	**<0.0001**
**Other opioids**	**2.2**	**<0.0001**	**1.9**	**<0.0001**
Female	1.0	0.41	0.9	0.17
Age 70–74	1.2	<0.0001	1.1	0.14
Age 75–79	1.6	<0.0001	1.4	<0.0001
Age 80–84	2.2	<0.0001	1.8	<0.0001
Age ≥85	2.7	<0.0001	2.2	<0.0001
Low income	1.1	0.0006	1.1	0.06
Medium income	1.1	0.05	1.1	0.08
Midwest	1.1	0.004	1.0	0.79
Northeast	1.1	0.001	1.1	0.05
West	1.1	0.006	1.1	0.29
PCP per 100,000	1.0	0.19	1.0	0.79
Medium coverage plan types	1.0	0.65	0.7	0.03
Other plan types	1.0	0.77	1.0	0.64
Antidepressants	1.4	<0.0001	1.6	<0.0001
NSAIDs	0.9	0.001	0.9	0.03
Physical therapy	1.0	0.45	1.1	0.01
Number CNS medications = 1	1.4	<0.0001	1.4	<0.0001
Number CNS medications = 2	1.8	<0.0001	1.7	<0.0001
Number CNS medications = ≥3	2.6	<0.0001	2.5	<0.0001

Reference categories included: no opioid use, age 65–69, high income, South region, high coverage plan types, no antidepressants, no NSAIDs, no physical therapy, and number of CNS medications = 0.

CNS medications, central nervous system medications (including benzodiazepines, non-benzo hypnotics, muscle relaxants, antipsychotics and gabapentinoids); NSAIDs, nonsteroidal anti-inflammatory drugs; PCP, primary care physician.

Cox proportional hazard regression analyses were used to determine the association of tramadol, other opioids, and no opioid use with falls/hip fractures, CVD hospitalizations, safety event hospitalizations, and all-cause mortality using variables in Table 2. Adjusted hazard ratios were calculated by opioid use categories comparing tramadol and other opioids to no opioid use categories with follow-up for time-to-event of up to 33 months. Subsequently, each category of opioid use was compared to each other level to establish statistical differences between categories.

## Results

Overall, 651,556 (51,994 new users, 19,945 continuing users, and 579,617 nonusers) met the inclusion and exclusion criteria described. After propensity matching, the equalized study populations consisted of 25,889 within each opioid use category (72% new users and 28% continuing users) (Table 1). New and continuing tramadol or other opioid users were relatively equalized on matching variables including age, sex, region, location, income, minority, access to care, plan type, and health status. Differences, however, were evident for increased selected medical conditions, use of other medications individually and in combination, and use of physical therapy in the post period for users compared to nonusers.

New users of tramadol were associated with an 80% increased risk of multiple ER visits and new other opioid users were associated with a 120% increased risk of multiple ER visits, compared to non-opioid users. Continuing tramadol users had a 50% increased risk and continuing other opioid users had a 90% increased risk, compared to non-opioid users (Table 2). Other variables strongly associated with multiple ER visits included use of ≥3 CNS medications, use of 2 CNS medications, ages ≥85 years, and ages 80–84. About 11%–16% of tramadol and other opioid users experienced multiple ER visits within the first year of follow-up compared to 7% for nonusers (Table 1).

**FIG. 1.**
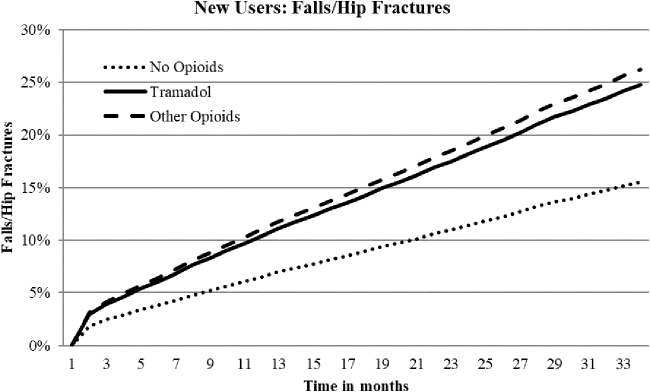
Adjusted time to event curves for falls/fractures for new tramadol and other opioid use vs. no opioids. Curves were regression adjusted with variables from Table 2. Tramadol vs. no opioids HR 1.59, *P* < 0.0001; other opioids vs. no opioids HR 1.69, *P* < 0.0001; Tramadol vs. other opioids HR 0.95, *P* = 0.02. HR, hazard ratio.

**FIG. 2.**
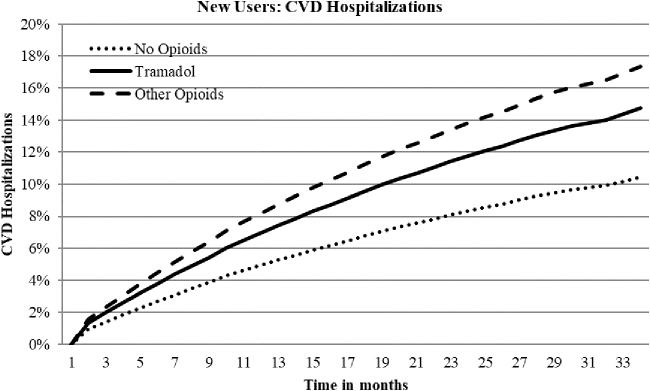
Adjusted time to event curves for CVD hospitalizations for new tramadol and other opioid use vs. no opioids. Curves were regression adjusted with variables from Table 2. Tramadol vs. no opioids HR 1.41, *P* < 0.0001; other opioids vs. no opioids HR 1.66, *P* < 0.0001; Tramadol vs. other opioids HR 0.85, *P* < 0.0001. CVD, cardiovascular disease; HR, hazard ratio.

**FIG. 3.**
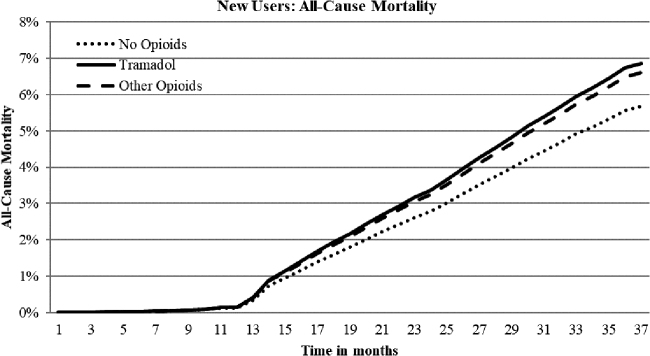
Adjusted time to event curves for all-cause mortality for new tramadol and other opioid use vs. no opioids. Curves were regression adjusted with variables from Table 2. Tramadol vs. no opioids HR 1.21, *P* < 0.0001; other opioids vs. no opioids HR 1.17, *P* = 0.0004; Tramadol vs. other opioids HR 0.96, not significant. HR, hazard ratio.

Hazard ratios (HRs) for falls/hip fractures, CVD hospitalization, safety event hospitalizations, and all-cause mortality for new tramadol use indicated a 59%, 41%, 35%, and 21% increased risk, respectively, compared to nonuse (Table 3; Figures 1, 2, and 3). New other opioids users were associated with a 69%, 66%, 55%, and 17% increased risk, respectively, compared to nonusers. Continuing tramadol and other opioid users showed similar, although somewhat reduced, levels of risk for each of the outcome measures with the exception of all-cause mortality for continuing tramadol users, which was not significantly different than nonusers (Table 3).

**Table 3.** Outcomes Associated with Propensity Matched New and Continuing Tramadol and Other Opioid Users Versus No Opioids

Outcomes	New users			Continuing users		
	Tramadol	Other opioids	No opioids	Tramadol	Other opioids	No opioids
Number	18,824	18,661	18,824	7,075	7,238	7,075
Unadjusted falls/hip fractures rates (%)	19.3%	20.9%	12.6%	21.3%	23.3%	12.7%
Adjusted falls/hip fractures rates (%)	24.8%	26.2%	15.5%	24.1%	24.8%	16.3%
Unadjusted CVD hospitalization rates (%)	12.0%	14.0%	9.0%	13.9%	17.6%	9.9%
Adjusted CVD hospitalization rates (%)	14.8%	17.4%	10.5%	15.0%	19.1%	11.5%
Unadjusted safety event hospitalization rates (%)	16.5%	18.9%	12.9%	19.3%	24.1%	13.9%
Adjusted safety event hospitalization rates (%)	21.4%	24.6%	15.8%	22.0%	27.4%	17.4%
Unadjusted all-cause mortality rates (%)	6.9%	6.7%	6.6%	8.0%	9.9%	7.2%
Adjusted all-cause mortality rates (%)	6.9%	6.6%	5.7%	6.6%	7.7%	6.5%
Falls/hip fractures, hazard ratio (95% CI)	1.59 (1.51–1.68)	1.69 (1.60–1.78)	Ref.	1.48 (1.35–1.61)	1.52 (1.39–1.66)	Ref.
CVD hospitalizations, hazard ratio (95% CI)	1.41 (1.32–1.51)	1.66 (1.55–1.77)	Ref.	1.31 (1.19–1.45)	1.67 (1.51–1.84)	Ref.
Safety event hospitalizations, hazard ratio (95% CI)	1.35 (1.28–1.43)	1.55 (1.47–1.64)	Ref.	1.27 (1.17–1.38)	1.58 (1.45–1.72)	Ref.
All-cause mortality, hazard ratio (95% CI)	1.21 (1.12–1.31)	1.17 (1.07–1.27)	Ref.	1.02 (0.90–1.15)	1.19 (1.06–1.35)	Ref.

All models regression adjusted for variables in Table 2. All hazard ratios for tramadol and other opioids users vs. nonusers significantly different *P* < 0.001 with the exception of all-cause mortality continuing tramadol vs. nonuser not significant. Hazard ratios for other opioids vs. tramadol significant for falls/hip fractures: new *P* = 0.02; continuing *P* = 0.04; CVD hospitalizations: new *P* < 0.0001; continuing *P* < 0.0001; safety event hospitalizations: new *P* < 0.0001`; continuing *P* < 0.0001; all-cause mortality: new not significant; continuing *P* = 0.004.

CI, confidence interval; CVD, cardiovascular.

About 25% of tramadol and other opioid users experienced a fall/hip fracture over the 33-month follow-up compared to about 16% for nonusers (Table 3). The prevalence rates for falls did not decrease over time for continuing users. About 15% and 22% of new or continuing tramadol users experienced CVD or safety event hospitalizations, respectively, compared to 17% and 25% for new users and 19% and 27% for continuing other opioid users. Prevalence rates for CVD or safety event hospitalizations increased with continued use. All-cause mortality rates were about 7% for new tramadol and other opioid users compared to 6% for nonusers. Over time, mortality rates for other opioid users remained stable; however, continuing tramadol users' rates decreased and were no longer significantly different than nonusers.

## Discussion

In this propensity-matched population of AARP Medicare Supplement insureds, about 72% were new users of either tramadol or other opioids; 28% were continuing users. Prevalence rates for potential side effects associated with pain or medications including insomnia, pneumonia, serotonin syndrome, and respiratory distress were significantly higher among tramadol and other opioid users compared to nonusers. Similarly, use of other medications often associated with pain management, such as antidepressants, benzodiazepines, and gabapentinoids, were associated with both tramadol and other opioid use. These characteristics may indicate inadequate pain management effectiveness, and/or complex patient characteristics necessitating additional medications to manage mental health and sleep symptoms. Generally, continuing users had similar profiles to new users for side effects and use of other medications with no evidence that longer term pain management had changed.

About 12% and 16% of new and continuing tramadol and other opioid users, respectively, used ≥3 ER visits in the 1 year following the tramadol or other opioid index date. Compared to no opioids, the risk of multiple ER visits is approximately doubled with tramadol or other opioid use. Although the specific reasons for the ER visits could not be determined, this measure appears to be a sensitive surrogate measure for care management needs. As expected, the risk for multiple ER visits for continuing users decreased somewhat compared to new users, but remained from 50% to 100% higher than for those with no opioids. Despite not being a strong cost driver for medical expenditures, multiple ER visits may provide a robust indication of unmet pain management needs.^9^

The prevalence ranges of CVD hospitalizations and the composite safety event hospitalization measure were about 14%–15% and 21%–22% for tramadol, respectively, and about 17%–19% and 25%–27% for other opioids, respectively. Although included, the prevalence of respiratory distress and serotonin syndrome from diagnosis codes was low (≤1%) and likely underreported. The HRs for new and continuing tramadol use indicated a 30%–40% increased risk for CVD or safety event hospitalizations. Other opioids demonstrated a 60%–70% increased risk. Prevalence rates remained stable or increased for new and continuing tramadol and other opioid users over the time period. These results are comparable to results reported by Solomon et al for overall opioids for specific CVD events (HR 1.77) and a composite safety event hospitalization measure including CVD, specified fractures, and gastrointestinal tract bleeding (HR 1.68).^16^ CVD hospitalizations generally are not included in opioid or tramadol adverse event studies; however, these results confirm previously published associations and warrant further study.

Falls/hip fractures are a consistent safety event reported in the literature for tramadol and other opioids use.^13,14,16–19^ Over the 33-month time period about 25% of tramadol or other opioid users experienced a fall/hip fracture compared to about 16% for nonusers. In the present study, the risks for new users of tramadol and other opioids were 60% and 70% increased, respectively. For continuing users, the risk for falls/hip fractures dissipated somewhat to 50% increased risk for both groups but, nevertheless, remained relevantly increased. Falls-related opioid research studies differ in study populations and time frames but generally demonstrate higher falls risk with initial exposure (eg, OR 5.60 first week for tramadol),^13^ decreasing to risk levels of about 30%–50% increased over longer periods of time (eg, 30 days or longer).^13,14,18^ Of note, regardless of study designs, falls risk levels for tramadol (when measured separately) and other opioids appear to be relatively similar.^13,14,18^ This would be contrary to the perception of negligible risk levels for injurious falls that might be expected for tramadol with longer-term use.

An established association of tramadol with increased risk of all-cause mortality has been considered only recently. Tramadol-related mortality may be related to seizures, respiratory distress, hypoglycemia, falls, or fractures.^11^ Present study results indicate a low level of mortality risk—about 20% increased for tramadol and other opioids similarly. Of note, however, although the risk for other opioids remained relatively stable for continuing users, the mortality risk for continuing tramadol users dissipated to nonuser levels with longer use. Present study mortality rates were lower than those reported in 2 recent research studies that documented an increased risk of about 70% for mortality associated with tramadol.^11,15^

An earlier study focusing on opioids indicated an 87% increased mortality risk associated with use.^16^ In this study, despite a robust propensity-matched comparison group, there remains a question of possible residual confounding in that patient characteristics may partially explain the results demonstrated. Nevertheless, although the magnitude of the association may vary across these separate studies, with notably different study designs, study populations, and time lines, there does seem to be a consistent association between both tramadol and other opioids with all-cause mortality, especially among new users.^11,15,16,26^

Overall, tramadol new and continuing users were associated with lower levels of risk compared to other opioids but consistently higher than opioid nonusers. The robustness of these associations across multiple outcome measures provides confidence that the risk for safety events associated with tramadol use warrants attention. Given the pharmaceutical choices for pain management available to primary care providers, tramadol may be an appropriate treatment providing a critical solution for patients within a broader pain management strategy.^6,32^ The side effects associated with NSAIDs, including gastrointestinal bleeding and risk of organ damage, limit long-term use of this class of drugs. Tramadol is not associated with gastrointestinal complications or organ damage and has a lower risk of respiratory distress compared to other opioids; thus, it has advantages to recommend its use.

Management of chronic pain requires individual solutions complicated by the number of pain sites, intensity of pain, mental health status, and sleep problems. Furthermore, reactions to various analgesics and/or drug combinations vary across patients. Although some patients tolerate drug choices and find pain relief, others do not. Thus, when accelerated pain treatment is required, tramadol fills an analgesic gap between NSAIDs and stronger opioids. Nevertheless, tramadol is not risk free and users experience more side effects and use more mental health and sleep medications than opioid nonusers.^2–5,9,14,15,17^

Implementation of regular medication reviews by physicians or pharmacists would facilitate the careful monitoring of dosages, inappropriate medication combinations, unnecessary medications, side effects, and effectiveness of pain management.^19^ Patient awareness of the composite safety of medication protocols could be enhanced with more comprehensive patient-provider communication.^16^ Given that most long-term opioid prescribing occurs in primary care, future research must focus on the development of more usable nonpharmacological interventions.^19,33^ To date, although such interventions exist, their availability to primary care providers has been limited.

### Limitations

This study has some limitations. The study population of AARP Medicare Supplement insureds may not generalize to all older adults or to those with other Medicare or Medicare Supplement plans. Prescription medication use was defined from administrative pharmacy databases reflecting purchase patterns but not necessarily consumption patterns. The researchers used robust propensity matched study populations but were limited to variables available within the administrative databases. Additional differences between the subgroups may exist for which the researchers did not have information, including self-reported cardiovascular risks and/or pain severity. Thus, despite propensity matching, residual confounding cannot be excluded as contributing to some outcomes. Strengths of the study include a large real-world community-based study population that included multiple safety event outcomes for new and continuing tramadol users and included comparisons with other opioids and no opioid subgroups.

## Conclusion

Overall, these results provide a systematic comparison of multiple safety event outcomes across new and continuing users of tramadol compared to new and continuing other opioid users and nonusers. Consistently, tramadol was associated with higher levels of safety event risks compared to nonuse. Thus, although tramadol may be prescribed as an appropriate analgesic within a broader pain management strategy for older adults with osteoarthritis, use of the medication is not without risk. Regular risk assessments along with careful monitoring for adverse safety events associated with tramadol use and more nonpharmaceutical options are warranted.
